# Etymologia: Orf

**DOI:** 10.3201/eid1901.ET1901

**Published:** 2013-01

**Keywords:** etymologia, orf, exanthematous infectious disease, viruses, contagious ecthyma, sore mouth, sheep, goats, parapoxvirus

## Orf [orf]

Origins of the term are unclear, but some sources (the Oxford English Dictionary, Webster’s) derive it from Old Norse *hrūfa* (“crust on a wound, scab”). Another source (Stedman’s) derives it from the Old English *orfcwealm* (“murrain, any infectious disease of livestock”), from *orf* (“cattle”) + *cwealm* (“destruction”). Paradoxically, although “orf” may trace its origin to a word meaning “cattle, orf does not naturally infect cattle.

Orf (contagious ecthyma) is an exanthematous infectious disease caused by a parapoxvirus that occurs primarily in sheep and goats but can be spread to humans through contact with an infected animal or fomites. Although most human cases are associated with occupational exposure to sheep or goats, cases have recently been reported in persons who were infected through household meat preparation or ritual sacrifice.
